# Central scotoma following phacoemulsification surgery: A case report on retinal phototoxicity

**DOI:** 10.1016/j.ajoc.2025.102334

**Published:** 2025-04-10

**Authors:** Tahmina Nazari, Maarten B. Jalink

**Affiliations:** a Department of Ophthalmology, University Medical Center Utrecht, Utrecht, the Netherlands; b Department of Ophthalmology, Central Military Hospital, Utrecht, the Netherlands

## Abstract

Phacoemulsification cataract surgery is a widely adopted, however despite its routine application and favorable outcomes, it is not devoid of risks. Complications are rare, with an even more infrequent occurrence of retinal phototoxicity. This case report delves into an instance of retinal phototoxicity in a 59-year-old Caucasian male following an uneventful phacoemulsification and intraocular lens implantation. Postoperatively, he experienced a small, central scotoma. Imaging revealed a small disruption in the outer retinal layers (subfoveal). In three months’ time, his central scotoma disappeared and the imaging showed improvement with only a minor outer retinal irregularity being present. This paper presents the case and discusses the risk factors to develop phototoxicity after phacoemulsification.

## 1 Case report

A 59-year-old Caucasian male presented with a small, central scotoma in his right eye immediately following an uneventful phacoemulsification and intraocular lens implantation (monofocal lens with a blue blocker, targeted at emmetropia). Total duration of the surgery was 13 min. No symptoms were reported in his left eye. The patient had a history of refractive laser (LASEK) treatment, with an initial refraction of −4.50 diopters. Before the phacoemulsification, his last spherical correction was −2.75 diopters in his right eye. Additionally, he had bilateral retinal detachments, both managed with pars plana vitrectomy. Patient is otherwise healthy and did not suffer from diabetes mellitus or other predisposing vascular diseases. The patient was not on any medication.

**Fig. 1:**
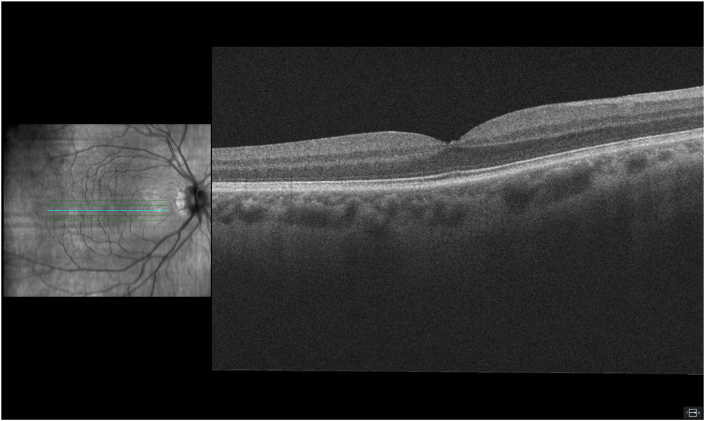
Preoperative OCT, showing a normal macula.

**Fig. 2:**
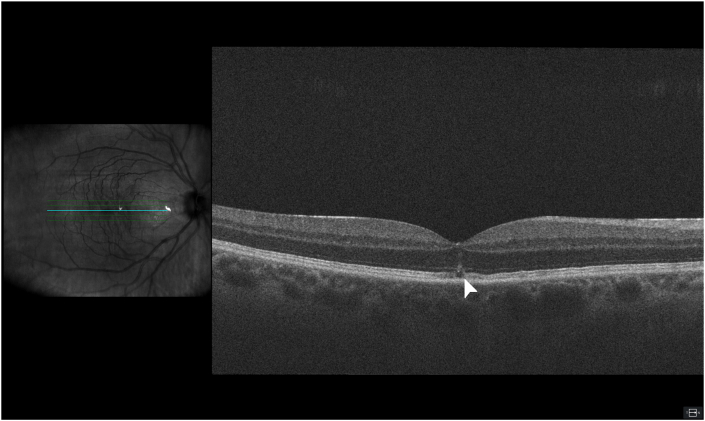
Macular OCT made 6 days postoperatively, showing disruption in the outer retinal layers of the fovea (white arrow).

Before the phacoemulsification, the visual acuity was 0.7 (Snellen), improving to 0.9 with a pinhole. Slit lamp examination revealed nuclear cataract, and fundus examination including optical coherence tomography (OCT) indicated normal findings (Fig. 1). Six days postoperatively, the patient reported a small central scotoma observed immediately after the procedure. He denied the use of any medication or drugs. Visual acuity at this point was 0.9 (Snellen) and both slit lamp and fundus examinations were unremarkable. OCT scan revealed a small disruption in the central outer retinal layers (Fig. 2). Watchful waiting was applied.

**Fig. 3:**
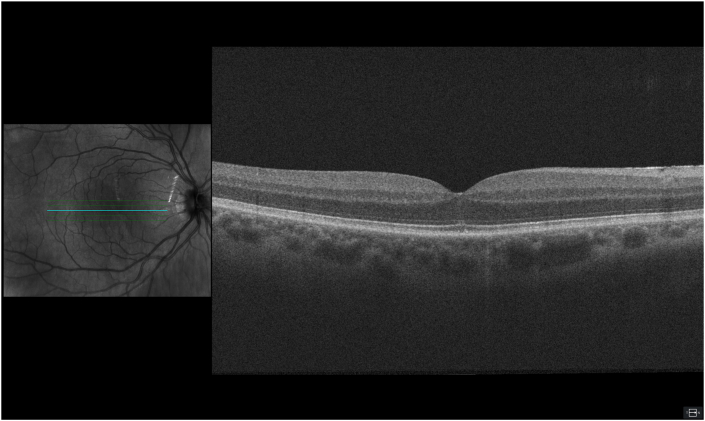
Macular OCT made 3 months postoperatively, showing spontaneous recovery.

Three months following the phacoemulsification of the right eye, the visual acuity improved to 1.2 (Snellen). Slit lamp and fundus examinations remained normal. The previous outer retinal disruption detected on the OCT scan improved, and only a minor irregularity in the outer retina was seen (Fig. 3).

## 2 Discussion

Phacoemulsification cataract surgery stands as the preferred method for cataract removal, as it is effective and provides a rapid postoperative recovery. This minimally invasive approach utilizes ultrasonic energy to emulsify and remove the cataract. Phacoemulsification combined with intraocular lens implantation, ranks among the most commonly performed surgeries globally, with low complication rates (1.2 %) and a high success rate (93 %).^1^ Despite its routine nature, phacoemulsification is not without risks. Complications like posterior capsule rupture and dropped nucleus can occur.^1^ Uncommon is the occurrence of retinal phototoxicity, a seldom-reported complication resulting from the intricate interaction between the light emitted by the operating microscope during surgery, the ocular structures, and the vulnerable retina.^2^ This report explores a case of retinal phototoxicity in a patient following phacoemulsification surgery.

Retinal phototoxicity can arise from exposure to intense light sources, such as sunlight (resulting in solar maculopathy) or, as observed in this case, the light emitted by an operating microscope, even during brief surgery.^2^ Phototoxicity following surgery is very rare and is influenced by various risk factors.^2^ Firstly, a relatively clear lens may contribute, as it absorbs less illumination during surgery. Secondly, a minor refractive error facilitates precise focusing of microscope light by the intraocular lens onto the retina. Additionally, smaller incision surgeries may pose a risk, as they cause less distortion of the ocular surface, allowing accurate focusing of the microscope light on the retina. Finally, light intensity settings should be kept at an acceptable minimum, as brighter light has a higher potential of causing phototoxicity,

Our patient presented with a visual acuity of 0.7 on the Snellen scale and nuclear cataract, demonstrating a relatively clear lens before surgery. Emmetropia after lens implantation acts as a risk factor for microscope-induced phototoxicity, as it focuses the microscope's parallel light on the fovea during the last part of the procedure.^2^ Furthermore, absence of residual astigmatism after refractive laser surgery minimized surface distortion, enhancing precise light focus. Lastly, the patient lacked vitreous following pars plana vitrectomy, allowing direct light exposure to the retina without vitreous deflection. The natural course of retinal phototoxicity may show favorable outcomes.^3^ Our patient's scotoma diminished, and visual acuity improved to 1.2 on the Snellen scale. Light intensity was kept at a minimum during the operation. A limitation of this report, however, is that this was not objectified as ophthalmic microscope (Lumera 700, Zeiss, Germany) settings are not reported after surgery.

## 3 Conclusion

In summary, this case report highlights a previously vitrectomized patient who developed a central scotoma following an uncomplicated phacoemulsification. While retinal phototoxicity is exceptionally rare, caution is warranted in patients with one or more risk factors.

## CRediT authorship contribution statement

**Tahmina Nazari:** Writing – original draft, Visualization, Investigation, Conceptualization. **Maarten B. Jalink:** Writing – review & editing, Visualization, Investigation, Conceptualization.

## Patient consent

Written consent from the patient regarding the publication of this case report and the associated images has been obtained and retained by the study authors.

## Funding

This study received no funding or grant support.

## Declaration of competing interest

The authors report there are no competing interests to declare.
